# Understanding System-Level Intervention Points to Support School Food and Nutrition Policy Implementation in Nova Scotia, Canada

**DOI:** 10.3390/ijerph16050712

**Published:** 2019-02-27

**Authors:** Jessie-Lee D. McIsaac, Rebecca Spencer, Melissa Stewart, Tarra Penney, Sara Brushett, Sara F.L. Kirk

**Affiliations:** 1 Healthy Populations Institute, Dalhousie University, PO Box 15000, Halifax, NS B3H 4R2, Canada; Jessie-Lee.McIsaac@msvu.ca (J.-L.D.M.); becky.spencer@dal.ca (R.S.); cmelissa.stewart@dal.ca (M.S.); s.brushett@dal.ca (S.B.); 2 Faculty of Education, Mount Saint Vincent University, 166 Bedford Highway, Halifax, NS B3M 2J6, Canada; 3 MRC Epidemiology Unit/CEDAR, University of Cambridge, Cambridge CB2 1TN, UK; tarra.penney@mrc-epid.cam.ac.uk; 4 School of Health and Human Performance, Faculty of Health, Dalhousie University, Halifax, NS B3H 4R2, Canada

**Keywords:** school food, complex systems, child and youth health, nutrition, environment, Intervention Level Framework, public health

## Abstract

Supporting the implementation of school food and nutrition policies (SFNPs) is an international priority to encourage healthier eating among children and youth. Such policies can improve equitable access, resources, and supports for healthy eating. However, despite the potential impact of SFNPs, several implementation barriers have been reported. This study sought to examine the system-level intervention points within a school food system using a complex systems framework. We conducted semi-structured interviews with various stakeholders working to influence the school food system in Nova Scotia, Canada. We sought to understand their roles and experiences with the SFNP by applying the Intervention Level Framework (ILF), a novel, solutions-oriented approach to better understand how complex systems function. Participants (n = 33) included teachers, parents, cafeteria workers, public health staff and non-profit organizations. Interview transcripts were first coded, then themed and finally analyzed using the ILF, resulting in three intervention points within the school food system. These were defined as: (1) Actors and Elements, (2) System Regulation and Interconnections and (3) Purpose and Values. We concluded that understanding the interactions between these system levels and stakeholder roles can help to inform the development of relevant policy strategies that better support healthier school food environments in this jurisdiction.

## 1. Introduction

Strategies that encourage healthy eating are an international priority to address diet quality among children and youth [1,2]. Over the past decade an increasing number of school jurisdictions internationally have advocated for, and introduced changes to, school food environments to support healthy eating [1,2]. These school food environments encompass policies and practices that influence the quality and types of foods served and/or sold in schools, as well as how food and nutrition is taught or modelled in these settings [1,2]. Healthy school food environments provide students with equitable access to healthy foods during the school day and support the attainment of nutrition knowledge and skills [1]. However, there are identified challenges with the implementation and sustainability of healthy school food environments due to factors such as a lack of leadership around policy implementation, inadequate resources, and incompatible social norms of unhealthy foods [1]. In particular, as schools are part of our communities, these broader system-level factors need to be better understood in the policy implementation process, as they represent “the interface where people interact with the wider food system to acquire and consume foods” [3]. This interface may include the interactions between various stakeholders, institutions, and organizations that influence the types of foods available to students, as well as the food-related social norms that operate within, and beyond, the school gates. However, identifying effective interventions to support healthy school food environments is a complex process that requires the consideration of a range of multi-faceted factors within the shifting contexts of education and health sectors [4,5]. Further, broader system factors also need to be considered, such as related industries, organizations, and government initiatives that influence our social norms and the foods available in our communities [2,3].

Systems-thinking recognizes the interrelationships and interactions across stakeholders, structures, processes, and contexts [6,7] and is particularly relevant for achieving systemic change within the education system [5,8]. Schools and education systems are complex, as they involve many interconnected elements, including, but not limited to, principals, teachers, families, communities, and infrastructure, that are organized to achieve multiple goals [9]. Research has used complex systems science in the context of the education system to explore various topics, including the social determinants of health [10] and achievement gaps [11,12]. Studies have also used complex systems science to explore food systems, with the majority of research to date focusing on interventions that address the obesogenic environment [13,14,15]. However, there is a dearth of research on how health and education systems interact to support or hinder student access to healthy foods.

**Figure 1:**
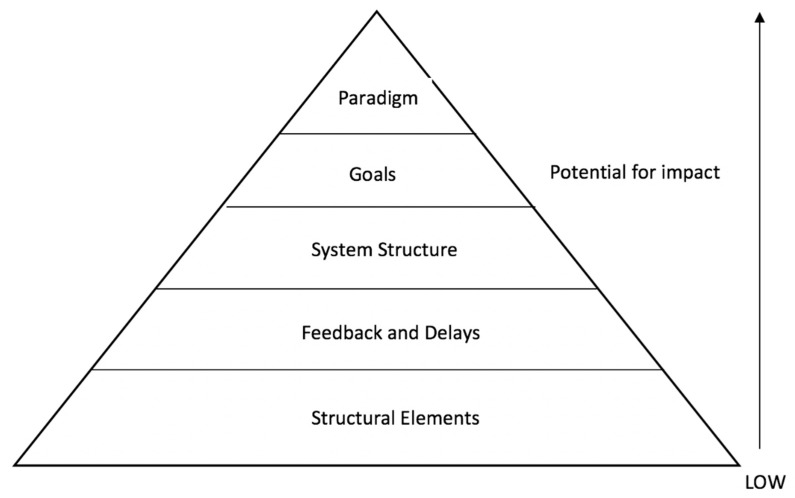
Intervention Level Framework (Johnston et al., 2014).

Multiple frameworks have been developed to support the application of complex systems science approaches to promote health or prevent chronic disease [16,17]. Typically, solutions to complex problems are considered through a reductionist lens that does not fully consider the elements and interactions within a broader system [18]. Meadows (2008) suggested that systems thinking is “*a set of elements or parts that is coherently organized and interconnected in a pattern or structure that produces a characteristic set of behaviours, often classified as its function or purpose*” and proposed 12 leverage points for effective intervention in systems [9]. To further support its application, the Intervention Level Framework (ILF) was developed by collapsing these points into five levels of operation for systems that offer expanding levels of impact on the overall system: Structural elements (potential for least impact), feedback and delays, system structure, goals, and paradigm (potential for most impact) [19]. In the context of school food systems, the application of the ILF extends existing literature related to food [20] and obesity [19], allows an exploration of the unique system interactions influencing school food environments and helps to identify opportunities for intervening. The five levels of the ILF are presented in Figure 1.

The province of Nova Scotia (NS) in Canada offers a unique case to explore system-level intervention points within the school food system because it has a rich history of research, policy, and grass-roots efforts to create healthy school environments [21]. Of note, a provincial school food and nutrition policy launched in 2006 has influenced the context of school food by mandating the foods and beverages permitted to be served and sold in schools as well as setting directives for pricing, programming and advertising, alongside guidelines that encourage schools to foster community partnerships and support local food products [21]. Among the first such provincial policy in Canada, the NS school food and nutrition policy comprises 12 directives related to the types of food and beverages served in schools, promotion and advertising, food and nutrition education, portion sizes and pricing, food safety and fundraising, alongside five guidelines related to time to eat, use of local produce and products, food packaging and environmental consciousness, role models and school partnerships and commitment [21]. Our past research has reported on the importance of school-level leadership, culture and organizational capacity to support implementation of the nutrition policy but has also highlighted the vulnerability of school actions to competing system-level priorities, financial limitations, and socio-cultural norms [22,23]. To support more effective implementation of the policy moving forward, the purpose of this study was to gain insight from key stakeholders involved in school food provision and policy implementation, and to examine the system-level intervention points in the school food system in NS through the application of the ILF framework.

## 2. Materials and Methods

Step one of this study involved collecting data through interviews with cafeteria workers, teachers, and parents of school-aged children in NS, and relevant employees of the provincial health authority. The purpose of these interviews, conducted in 2016–2017, was to gather more in-depth information about stakeholder roles and perspectives on healthy school food environments, including factors that may obstruct or enable actions to create them. Participants were purposively recruited via multiple recruitment strategies that included posters, social media, word of mouth, and snowball recruitment strategies, and included internal and external stakeholders, such as food distributors and relevant non-profit organizations. Initial contact with individuals was made by e-mail and phone, whereby participants could review the study details and respond confirming interest, date and location for the interview. A small number of individuals did not respond to invitations for interviews or were unable to confirm a time. One individual declined to participate due to illness. Ethical approval was provided by the Dalhousie University Research Ethics Board. Informed consent was achieved verbally for each phone participant and written or verbally for in-person interviews. Semi-structured interviews were conducted using an interview guide including broad questions designed by the research team to facilitate the interview, that was untested prior to this study (see Table 1). Twenty-six of the interviews were conducted over the phone and eight were conducted in-person at a location of the participants’ choosing. Interviews lasted between 45–60 min and all but one (at the participant’s request) were audio-recorded with consent. Interviews were conducted by authors R.S. and M.S. Due to external factors influencing the research (industrial action within the school system), public health interviews took place in spring/summer of 2016 while all other interviews took place in spring/summer of 2017. Our only inclusion criterion was participants having involvement with, or an interest in, school food provision, with a focus on the engagement of multiple stakeholders to ensure diversity in participation. There were no specific exclusion criteria applied.

In total, 34 interviews were conducted, of which 9 were teachers, 8 were staff working with the provincial public health system that has the responsibility for food and nutrition policy implementation within the school system, 8 were parents of students within the public school system, 5 were school cafeteria staff and 4 were external stakeholders involved in aspects of food distribution or resource provision in schools, as noted above. There was one repeat interview where the same individual was interviewed twice, once as a public health staff member during the first wave of interviews in 2016 and again as a parent in 2017, resulting in 33 total participants. Of the 33 participants, there was representation across all four public health regions that are operational in Nova Scotia, and half of the seven provincial school boards, which represent the administrative structure for school operations in the province. A small number of participants had a prior relationship with some members of the research team, particularly those in the external stakeholder group. In these cases, the interviewer with the least amount of association with the participant conducted the interview. In-person interviews were conducted at locations selected by the participant and included their homes, offices or rooms at the office of the researcher. Most interviews were private conversations between the participant and interviewer, however some participants completed interviews with children, students or co-workers within audible proximity. Proximity of others, especially co-workers, may have influenced what participants shared, however allowing participants to participate at times and locations convenient to them was important in making the study more accessible to busy and hard to reach populations (e.g., parents and cafeteria staff).

Step two of the study involved the application of Qualitative Description (QD) methodology to better understand the interaction between system levels and stakeholder roles in the complex school food environment. Being less interpretative than other designs, QD has the purpose of understanding and richly describing participant experience, particularly in understudied areas, and allows research to remain close to the data, permitting description of phenomena and events “in the everyday terms of those events [24,25]. Audio-recordings were transcribed verbatim and were imported into NVivo (QSR International Version 11) software for data organization, coding, and thematic analysis [26,27]. Data analysis was conducted primarily by two research assistants (S.B. and M.S.), guided by a member of the research team (R.S.). Open coding was conducted for each interview, with each person initially coding three interviews independently, recording comments and memos, prior to comparing. Frequent meetings were held to discuss emerging codes, questions, and concerns. Following coding, S.B. and M.S. engaged in thematic analysis, again under the guidance of R.S. In this phase, themes and subthemes were developed that described the interactions between stakeholders involved or interested in school food provision, and the stakeholder roles within this environment. Participants were not involved in reviewing transcripts or analysis, however multiple steps were taken to ensure the data represented the participants’ true meaning as accurately as possible. Researchers were reflexive while coding, actively recognizing the biases that they each brought to the data and field notes and peer review through frequent meetings and joint coding contributed to the credibility, authenticity, and dependability of data [28]. Data saturation was determined to be reached when no new themes emerged from interviews [27].

Step three of this study applied the ILF framework to the themed data so that participant experience could be understood with a view to elucidating potential system-level intervention points to inform future policy implementation, based on the definitions provided by Johnston et al. (2014) (see Figure 1). Given the focus of the ILF on levels of intervention, we felt this framework could uncover potential points of interventions that would help to achieve system-level change in Nova Scotia’s school food environment. To do this, we mapped the definitions and descriptions from the five levels of the ILF to our thematic data. Presentation of themes and data, including unidentified participant quotations, were determined by lead authors J.D.M. and R.S.

## 3. Results

**Figure 2:**
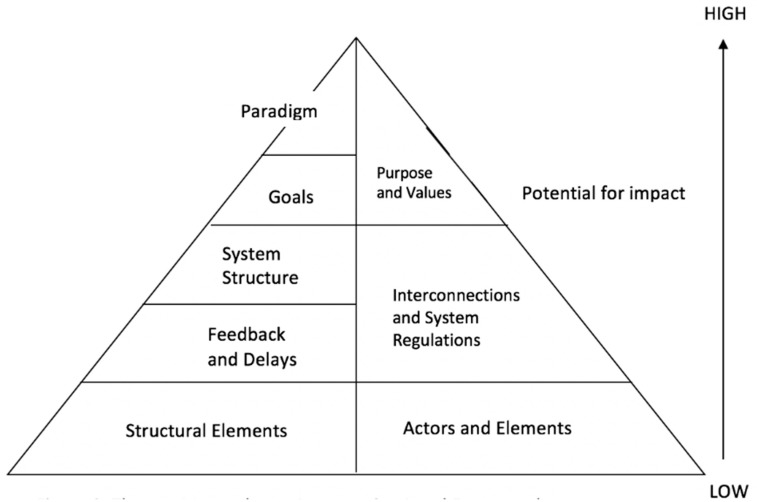
Themes mapped onto the Intervention Level Framework.

When mapped to the ILF, the qualitative data generated in this research resulted in three themes that could be considered as system-level intervention points (see Figure 2). The first theme we identified, Actors and Elements, aligned with the lowest level of the ILF: the structural elements. The second theme we identified, System Regulation and Interconnections, aligned with the middle levels of the ILF: feedback and delays, and system structure. The final theme we identified, Purpose and Values, aligned with the highest levels of the ILF framework: g\Goals and paradigm.

### 3.1. Theme 1: Actors and Elements

This theme relates to the structural elements and pieces of the school food system, aligned with the lowest level of the ILF framework, and characterized by the physical elements and the presence (or absence) of different actors within a system. Actors identified in this study consisted of students, families, teachers, principals, cafeteria workers, food distributors, school boards, government, and a variety of external stakeholders like community organizations and retail or fast food outlets. At this level of the ILF, actors were seen to be either present or absent from discussions related to school food policy implementation, or to have specific roles and responsibilities related to it. The majority of these discussions related to the actors’ impact on the creation and maintenance of school food systems. The interactions, perceptions, and mechanisms for communication among actors represent a higher level of the ILF and are therefore further discussed in the themes below.

In relation to system elements, two subthemes emerged, which were Resources and Finances and Funding (Table 2), which further highlight the variety and importance of various system elements. Resources of many types were frequently discussed by participants, often representing an aspect of constraint and limitation. One of the most salient resources described was time. A cafeteria employee, noted, “*when you’re serving that many kids, you can’t probably make your muffins from scratch every day. It’s got to be something that can be prepared quickly*”. This closely connected to the availability of staff and volunteer capacity. A teacher said, for example, in reference to expanding a breakfast program, “*I just, I’m the only one who does it, so just no human power, there’s no manpower to do it, I just don’t know if it’s feasible*”, while a public health employee described the school food policy as “*something else added to their (the school’s) plate and it’s a plate that’s already full*”. Other resources discussed by stakeholders as necessary were related to education and training, for food providers and distributors, cafeteria staff, and teachers. One external stakeholder summarized this by listing opportunities needed to increase the capacity for supporting a healthy school food environment, including, “*sharing best practices, creating a community of practice, supporting stories, reimagining budgets, tools, training sessions*”.

Kitchen space and equipment also emerged as important resources, relating to the ability to serve and store healthy food items. This connected both to school operations, demonstrated by an external stakeholder asking, “*how are you supposed to feed anywhere between 100 and 400 kids if you don’t have the space to hold the food?*”, as well as family experience, as parents discussed the challenges of not being able to send food that required refrigerating or being heated. Finally, participants described local community organizations as a positive resource for schools by helping with breakfast programs and developing school gardens, for example. Valuable community resources included local farmers, which was felt to facilitate, “*a different tone around food - kids know those farmers in their community*”, as well as local organizations like clubs and churches, that hosted breakfast and lunches.

As part of this theme, and closely connected to resources, was the subtheme related to finances and funding. Finances, money, and funding were described as important structural elements in various ways and from a variety of perspectives, though frequently as a challenge or constraint. Stakeholders broadly discussed the overall amount of available funding as limited, with some emphasizing the lack of federal funding for a national school food strategy. One parent noted: “*…you know, have the funds available from the government whether it be local level or federal level to be able to put some type of lunch program in place for all of our kids, not only for the kids who would be able to afford it but for all the kids in Canada*”. Fundraising was also discussed, noting the historical tendencies of schools to sell unhealthy foods to raise money, and the limited alternative options. Others highlighted the need for increased cost savings within school cafeterias that move toward healthier foods, noting staff hours might be cut to save money, and the impact of healthy food on operating budgets. One cafeteria employee said, “*like before the profits were high but it wasn’t healthy food because as you know, when you buy healthy you spend more, but I find that it’s not as important to make a profit as it is to provide the service*”. Finally, stakeholders connected funding to human resources, with one external stakeholder saying, “*the funding piece will go hand-in-hand with more human resources, there’s only two boards in our province that have actual dietitians working for them - we could use a lot more*”.

### 3.2. Theme 2: System Regulation and Interconnections

This theme aligns with the middle levels of the ILF framework, connecting to feedback and delays, or system regulation, and system structures, or the interconnections between system elements. This theme is also illustrated through several subthemes including first, interacting roles and responsibilities, second, food access, and third, communication, collaboration, and direction (Table 2).

The first subtheme related to system interconnections and regulation illustrates the interacting roles and responsibilities of the various system stakeholders and their interdependence with other system elements. Families were discussed by most stakeholders as having the biggest influence on students through their roles as primary food providers and role models for healthy or unhealthy food behaviours. Parents that participated in this research demonstrated varying degrees of understanding of school food systems and nutrition policies but they unanimously supported creating healthier school food environments. Students were also identified as having an important role in system regulation as consumers of school food and potential agents of change, although stakeholders noted that they were often overlooked. A teacher said, for example, “*I think you should talk to the students themselves - they would definitely have opinions on food at their school*”. Student preference was also highlighted by stakeholders. In some participants there existed a perception that youth do not like healthy food. Other participants identified this as misperception. One cafeteria employee noted that: “*the kids nowadays like the healthy food, before it was a struggle. they know what they want now and they want healthy*”.

The role of school staff was also discussed in relation to system regulation. Principals were highlighted as having a particularly important role in school leadership, related to setting direction and tone. One stakeholder said, for example, “*there’s some schools where the principal was very involved and very concerned about the food and there’s other schools where they’re not. And so that’s what creates the challenges*”. Teachers saw their responsibility primarily around role modelling, with one surmising, “*Practice what you preach. I’ve always allowed my kids if they want to have a snack in class, it’s no problem, but it has to be a healthy snack*”. Cafeteria staff also played an important role in the regulation of school food systems; participants discussed their role in what food is served and sold, how it is prepared, and how their knowledge, skills, and relationships with students influence cafeteria operations, although many participants noted that cafeteria staff are typically undervalued and underfunded. One cafeteria employee described their relationship with students, saying, “*I really get to know the kids really well and so I ask them you know how was that today, did you like that, do you think I should do this again?*”.

Beyond schools, further interconnections and system regulations involved school boards/districts, government, communities, and other external stakeholders. Participants noted that the role of the school board was complex, and that more collaboration with government was required to facilitate healthy school food environments. Food distributors and suppliers were highlighted as an important interconnection for school food systems. One external stakeholder noted: “*some schools are a little more lax then others—we’re just here to give them what they’re asking for, they order it, we supply it*”. This was furthered by a public health employee who noted challenges around distributors’ “*interpretation of the policy and the advice they give to schools*”. As previously mentioned, community assets and organizations, like farms and non-profit organizations were seen as valuable connections by several stakeholders, though challenges around “*red tape*” in accessing schools were also noted, such as by one parent, who said, “*I get the sense though that the schools aren’t necessarily as open to support from the broader community, or that there’s barriers to them doing that*”. This also relates to the below sub-theme of access in reference to the bureaucracy that limits partnerships with schools. Finally, many discussed the availability of unhealthy food from retail and fast food establishments, noting that while healthy food may be available in schools, students could choose to eat in their communities instead. This competition between school cafeterias and retail outlets was highlighted by a public health employee who said, “*with zoning bylaws, I think we could really see an increase in school food if we could decrease the amount of fast food restaurants that are around schools*”.

The second subtheme within system interconnections and regulation relates to food access which was discussed in a variety of ways by stakeholders, particularly given the role of the school food and nutrition policy in shaping food access. Some stakeholders discussed the challenges of food access in relation to socioeconomic factors. This was seen to be more influential on students’ health than the system elements previously discussed. One teacher noted how, “*I also have worked with kids of so many different socio-economic, various backgrounds and there’s so many different factors that come into play. Food security is a huge issue and fresh fruits and vegetables for some people is just not an option all the time*”. Broader regulatory changes in the school food system over time also connected to access, with one cafeteria staff member saying, about students, “*they know that they’re not going to get hotdogs at school*”, and another stakeholder saying, “*we no longer have deep fat fryers or pop machines, and those types of things in schools*”. Similarly, access was connected to cost, with one parent saying, “*I don’t know if there’s anything that can be done to lower food prices but—it’s shocking how expensive food is*”. Proximity to retail food environments was also discussed as facilitating access to unhealthy food. Convenience was further discussed by most stakeholders, regarding fast food chains, in addition to packaged foods that are easy to store, and have long shelf lives, making them easy for students to access.

The final subtheme related to interconnections and system regulation was communication, collaboration and direction. All stakeholders discussed the idea of collaboration being required to support healthy school food environments, noting that stakeholders, such as boards, principals, school staff, and families need to work together or toward the same ideas in order to see the most impact. That said, many also discussed challenges in role clarity, responsibility, and resources, which limited their ability to work collaboratively. A public health employee said, “*it’s really a multi-pronged approach. You know, it’s really collaboration and partnerships because again, the schools have a lot on their plate*”. The idea of communication was also closely tied to collaboration, such that it is a necessary part of relationship building and maintenance. A teacher described a challenge presented by communication, saying, “*the functionality of the cafeteria is completely unknown to the staff. And I’ve gone in, ‘how do you decide that chicken fingers are going to be served on Thursday?’ The workers there are also not too sure, and I’m wondering if it’s more of a, you know, coming from a head office*”.

A parent further highlighted the role of communication in discussing elements that make it easier for schools to provide healthier food environments, saying, “*supportive leadership, working as a team, and being really kind of positive and open to talking. I always find connecting like being able to talk or dispel any kind of notions - so more ways to connect*”. Participants indicated that, in order to collaborate, there needs to be communication, and with communication comes clarity in direction. The idea of direction was discussed by many stakeholders, often in terms of waiting for or needing instruction or clarity, and also in relation to ideas around autonomy and flexibility in their roles. A cafeteria employee said for example, “*I sort of do my own thing. I don’t, like nobody comes down and says you should do this, you should do that, they know that I know what we should do*”. This autonomy was generally preferred by cafeteria employees but they also identified the need for support from others, particularly related to their budgets and resources. Several stakeholders noted that changes to school food environments, including the nutrition policy, required more direction and leadership, with one parent illustrating this by saying, “*I could be pleasantly surprised, maybe they (the school) do talk about the policy and promote it, that would be great but I would guess that they don’t do it much*”.

### 3.3. Theme 3: Purpose and Values

The theme of purpose and values aligns with the highest levels of the ILF framework, relating to goals and paradigms, and illustrates a system’s philosophies, commitment, and beliefs. This level offers the most in terms of potential for impact but is typically the most challenging to influence. This theme was also illustrated by several subthemes, including control, disconnect and misalignment, competing priorities, and food culture (Table 2).

The concept of control emerged frequently through the exploration of the multiple levels of the school food system. Ideas around who can and should control the food choices of children, who controls how and where funding is spent, and how school food policies should be managed and implemented were commonly discussed by stakeholders. Alongside the idea of control, was that of accountability, where participants discussed whose responsibility it might, or should, be to ensure a healthy school food environment. Further to this, was the concept of individual versus collective responsibility and having a balance between individual control and autonomy and caring for our children societally. A teacher said, for example, “*we’re educators, you know, we need to inform kids on proper choices, healthy choices but at the same time they need to be able to be given that, the choice to make that choice*”. A parent illustrated these competing ideological challenges saying, “*I know that one of the people on the (Parent Teacher Association) said, ‘well it’s not for us to be the role models for these kids, it’s the parents to do that’, which I disagree with, I think the school should be a role model. I think there’s a lot of that; that people are free to choose what they want to do, you know, it’s a free society*”.

A second subtheme, disconnect and misalignment, illustrated the wide variety of perspectives and priorities relating to food and food environments that were held by various stakeholders. Participants discussed how priorities, mandates and perceptions map onto divided jurisdictions with varying capacity and resources, creating a challenging context in which to support healthy food environments consistently. A parent described this complex context saying, “*it’s about more than just what happens at lunch time - I think it needs to be more holistic. Ideally there would be pieces of curriculum that would reflect what they’re seeing at the cafeteria level and then what they’re being role modelled by staff and then there would be a policy that also supports that and the environment physically would be one that’s free of harmful influences and then the social environment would then follow suit and you know people would have those positive feelings about food*”. Another parent noted the autonomy of individual schools, “*I think we’re siloed still too much, each school doing its own thing which makes it just subject to the people that are there*”.

Ideas around disconnect and misalignment were also closely connected to the third subtheme of competing priorities. Stakeholders discussed challenges around cafeterias operating as for-profit businesses, and teachers and principals having workloads such that supporting a healthy food environment was perceived to be in direct competition of other priorities. A public health employee described one such challenge, “*our principals, with everything else, they’re concerned about keeping their food service workers employed. Not everyone has a business degree, the message around healthy school food environment, (it) can get lost in the management of the food service*”, while another echoed, “*it’s kind of a catch 22, it would be really nice to figure out what is the amount of time that’s required to provide, to prepare quality healthy foods that kids will eat and that you can make money*”.

As a final subtheme relating to purpose and values, food culture emerged as an important concept that illustrated participants’ understanding of the deeply held beliefs, perspectives, and norms relating to food and how we interact with it both societally and in the school food context. Many stakeholders discussed the important influence of advertising and marketing, and how media influences youth. One parent summarized by saying, “*I know that a lot of companies market directly to kids and that’s kind of, there’s some ethical questions about that, I think some pretty dodgy marketing going on and, it would be nice if that was addressed and regulated*”. Others discussed the concept of “treats” and special occasions, which become so frequent in the school environment that they are no longer occasional or special; one stakeholder said, “*broadly there is more of a movement toward healthy eating and valuing healthy food but there is this kind of counter balance ‘but like oh this is a special occasion’, and it’s just when the special treats become everyday things*”. There were also many examples of food culture connected to how food is treated in schools, such as one parent, who described eating at school to be perceived as, “*a nuisance to the school culture, it’s disruptive, it’s you know it’s something that, it’s only done at a certain time of day. I think the whole culture is just so backwards*”. Others discussed the lunch time at schools, which has several activities, priorities, and circumstances happening simultaneously, speaking to whether, or how time for nutrition was valued. A public health employee illustrated this perspective, saying, “*the culture of lunch time at the schools is an issue. Students are leaving for many reasons, I don’t think it’s necessarily to get unhealthy food, I think it’s like the social pressure of being cool enough to leave school. It’s the culture of what’s in a cafeteria and what the cafeteria looks like, and it’s the culture that schools are starting to lose intramurals which is what is keeping them at school*”. Several participants also discussed food culture from an employment perspective, in that those who work in food service tend to be societally undervalued, with one stakeholder saying, “*if we’re going to talk about health, we have to talk about equity, like when I think about what food service workers make in comparison to teachers—I don’t know why we would value the people who feed us so much less*”. Some stakeholders discussed education as a potential solution to many of these issues, and while others thought that would be sufficient, others highlighted that education should be paired with resources, salaries, time, and broader commitments to valuing and supporting healthy food environments.

## 4. Discussion

This study examined system level intervention points across the school food system, derived from interviews with key stakeholder groups in NS, Canada. Using the ILF, we aimed to go beyond a conceptual consideration of the complexity within school food environments that suggest simple solutions and that assume rationality and linearity [29,30]. Rather, the ILF framework provided an opportunity to unearth the interactions within the system to identify opportunities to intervene and create a healthier school food system. Through this process we identified three system-level intervention points within the provincial school food environment, and gained a better understanding of the role of different stakeholders within this system. These themes together corresponded with the five leverage points in the ILF, although they did not map exactly to the five levels of operation, thereby suggesting different opportunities to intervene on the school food environment in this provincial context. We also identified interactions between the emerging themes, which align with a system lens that considers the interdependence of factors [18]. For example, in this study, “time” was discussed as a resource, but it also related to the misalignment or disconnect between the value of nutrition in schools. Advertising and social marketing was another example, with labeling on vending machines identified as a conflicting resource in schools while also being identified for its contribution to the food culture within schools.

The theme that related to the highest leverage point in the system, goals and paradigms, highlighted the importance of alignment in priorities and food culture in the creation of a healthier school food system. Conflicting goals related to nutrition in schools is commonly reported as a barrier to improving school food environments [31,32,33,34,35,36,37] considering the prioritized mandates and prevailing paradigm that operates within schools toward traditional educational goals [38]. Focusing on concrete actions at the goal-setting level, such as the adequate and consistent enforcement of the school nutrition policy, would provide feedback to shift the dynamic of the system toward significant change [19]. Broader societal norms and values that operate outside of the school food system inevitably shape the norms and values of food in schools [38,39] and challenge policy implementation, as has been noted in previous research [22,23]. Although difficult to shift, framing actions to align with the school’s purpose and values, which also aligns with the “paradigm” level of the ILF, the highest leverage point within a system, will be important to re-frame and normalize a new way to think about food within schools. An example of where this can be applied is in the ‘treats and celebrations’ narrative, where participants highlighted the multiple occasions where low-nutrition foods are available within schools, thereby challenging adherence to the policy and undermining the attainment of a supportive food environment. Ostensibly as a “treat”, these foods were so readily and frequently available that they were considered normative. Although participants did not wish to prohibit these types of foods entirely, most found their excessive availability contradicted healthy eating goals. Previous research has reported the presence of low-nutrient, energy dense foods through classroom celebrations [40,41] and the importance of school policies and engaging key stakeholders in strategies to shift norms and expectations related to celebration foods [42,43]. The emergence and success of healthy fundraising initiatives, such as fundraising through the provision of local produce boxes or non-food related options, is an example of positive change, where previously the norm has been for schools to rely on low-nutrition foods for fundraising initiatives [44,45].

The second theme focused on interconnections and system regulation, specifically on roles and responsibilities, access and convenience, and communication, collaboration and direction. This theme aligns with feedback and delays and system structures, highlighting the complexities of the school food system and the communications gaps among stakeholders. As highlighted in our results, stakeholders at each level of the system had their own perceptions of who was responsible for supporting healthy school food environments and who should be connecting with whom to make it happen. Almost all cafeteria staff interviewed spoke about positive relationships with their school principals and nutrition-related support but felt isolated from board-level decisions. Though they are tasked with delivering many elements of the policy and making budgets work, cafeteria workers expressed instances of not feeling supported, often spending extra time to find the best deals to source food. These challenges for cafeteria staff are noted in recent research exploring their perceptions, which highlights challenges around support and communication [46]. This offers an important intervention point so as to ensure that all relevant stakeholders are engaged in decisions about school food, not just the “usual suspects”.

Many participants spoke about their experiences with contracted distributors, suppliers and food service management contracts, highlighting the influence they have on the school food environment and tensions that exists among some stakeholders. The role of distributors in farm-to-school programs has been explored by some authors, revealing structural limitations in the types of foods that can be provided, the locality of the food and the packaging in which it is distributed [47]. To our knowledge there is no research specifically exploring the relationship of food distributors and school cafeterias in a Canadian or Nova Scotian context, however, it is clear from participants that this relationship is important and needs to be considered in conversations about improving the school food environment. Principals acted as connectors from the board level to the cafeteria, but the quality of this connection was dependent on the principal’s capacity and willingness to support school food. The voice of youth was highlighted as something missing from as a mechanism for feedback within the system from all stakeholder groups. Despite being the main reason for creating healthier food environments, other research in NS has affirmed that youth are often not consulted on decisions that will impact their immediate environments and food options in schools [48]. Although not reported in this paper, our research has also engaged youth to ensure their perspective is included (data not shown). We chose not to include them in this analysis because traditional interviews are not necessarily the best way to engage youth [48]. Therefore, we selected a separate methodology (photovoice) more suited to typically marginalized voices, such as youth, but this difference in methodology makes it more challenging to apply the ILF in the same way as was possible with the interviews described in this paper.

Finally, the physical elements in a system, including resources, funding and time was identified as the lowest leverage point within the system, but are often the most frequently acted upon. Although these elements are closely connected to larger influences related to values and priorities, solely focusing on action at this level would offer a simple solution to a complex problem. Specific resources noted in other studies as being critically important for supporting a healthy school food environment include lesson plans, materials and expert assistance for nutrition education [31,49,50,51,52,53]. While individually, these resources are more focused on structural issues, if implemented in a coordinated way, they could produce a shift in the system toward a healthier school food environment [9,54].

The strengths of this study include the use of a complex system lens and the variety of stakeholder roles that were included. We also connected system aspects to stakeholders, which is an important contribution to the literature. A key limitation is that some perspectives may have been missing from the data. We sought to gather a variety of perspectives of stakeholders involved with supporting school food in NS, with participants recruited through multiple recruitment strategies, but they may still represent a selected group of those that are interested in the issue. It is important to note that several attempts were made to involve a greater number of food distributors and service providers. However, these perspectives were under-represented and further study is needed with this stakeholder group. In addition, student voices were not part of this study, although as noted above, they are engaged in additional aspects of the broader research project. Qualitative work is inherently biased, and as health researchers, the interviewers were personally interested in promoting healthier school food environments. These biases were mitigated through regular meetings to discuss observations, biases, challenges and whether data saturation was being reached.

## 5. Conclusions

Creating a healthy school food environment means targeting features of the school that may support healthier eating among students, such as access to healthy foods, programs or initiatives and creating a culture where healthy eating is modeled and supported throughout the school day. This paper examined the system level intervention points across the school food system in the province of NS and from the perspective of key stakeholder groups [19]. We used the ILF framework to better understand the complexity of the school food system [9]. We identified three themes or intervention points within the school food system that offer guidance to decision-makers provincially and elsewhere. At the highest leverage point were the broad goals and paradigms, or purpose and values, that offer the potential to shift thinking to better support healthy food provision in schools. This can be enhanced by understanding the system regulation and interconnections and physical system elements operating at a lower level. These themes, which were found to be interconnected and interdependent, highlight the complexity of the school food system, and are in turn influenced by the various actors and elements that represent the lowest level of intervention. Taken together, this research highlights the importance of aligning these system actors and elements, and the value of targeting the highest leverage points in the system, such that a healthy school food system might be more consistently supported.

## Figures and Tables

**Table 1:** Interview questions used in this study.

Q1	Can you briefly describe how you support healthy school food environments in your current role?
Q2	Can you describe how your role as a food provider aims to support the implementation of the school food and nutrition policy?
Q3	Based on your experience, what prevents schools from creating a healthy food environment?
Q4	Based on your experience, what makes it easier for schools to create a healthy school food environment?
Q5	Who/what are the other important people/organizations/factors that influence healthy school food environments?
Q6	How could a revision of the NS school food and nutrition policy influence your work/role?
Q7	Who else should we talk to?

**Table 2:** Descriptions of themes and subthemes identified within the school food system.

Theme	Subtheme
**1: Actors and Elements** The human, material and monetary resources present or lacking in the school food system, as identified by participants.	Resources: The available human capacity and materials for school food stakeholders to implement healthy eating practices within school food environments **Time** **Staff/Human Resources** **Education** **Kitchen Space and Equipment** **Community**
	**Finances and funding: The costs, prices, profits, fundraising and funding opportunities available associated with school healthy eating programs.** **Funding available** **Cafeterias as businesses** **Fundraising** **Human resources**
**2: Interconnections and System Regulation** The interactions across the network of actors/elements and system structures (e.g., policies) and their influence on the school food environment.	Interacting roles and responsibilities of actors: Understanding of stakeholder positions, obligations, jobs, duties and priorities and their intersections across the system. Parents & students School staff School boards & government external stakeholders
	Food access: The degree and variety to which foods (healthy and unhealthy) are offered and the ease to which students, families and schools can acquire those foods. **Socioeconomic factors** **Regulatory changes** **Cost of healthy foods** **Retail food environments** **Convenience**
	Communication, Collaboration and Direction: Referring to the clarity of information transmission across institutions and stakeholders, particularly with regards to organizational and personal mandates and duties. Role clarity and responsibility Communication and relationships Clarity of direction
**3: Purpose and Values** The beliefs, attitudes and patterns that motivate behaviors and influence decision-making and their impact on school food systems.	**Control: Having or lacking power over food choices and decisions.** Responsibility for student food choices Accountability of school food Individual versus collective responsibility
	**Disconnect and misalignment: Incongruity/alignment between institutions and/or stakeholders’ agendas, priorities, mandates, understandings, etc. across jurisdictions.** Differing priorities, mandates and perceptions School autonomy
	**Competing priorities: Daily issues activities, ideas, agendas, events, and goals that have varying degrees of importance, precedence and emergence in the school food environment.** Cafeterias operating for profit Heavy school staff workloads
	**Food culture: People’s deeply held beliefs and perspectives about food and how we interact with it.** Deeply held beliefs, perspectives and norms of food Advertising and marketing Special occasion treats School culture
